# Phenylbutyrate, a branched‐chain amino acid keto dehydrogenase activator, promotes branched‐chain amino acid metabolism and induces muscle catabolism in C2C12 cells

**DOI:** 10.1113/EP089223

**Published:** 2021-01-20

**Authors:** Hannah Crossland, Kenneth Smith, Iskandar Idris, Bethan E. Phillips, Philip J Atherton, Daniel J Wilkinson

**Affiliations:** ^1^ MRC‐Versus Arthritis Centre for Musculoskeletal Ageing Research National Institute for Health Research (NIHR) Biomedical Research Centre (BRC) Clinical, Metabolic and Molecular Physiology University of Nottingham Royal Derby Hospital Derby UK

**Keywords:** branched‐chain amino acids, mTOR, muscle protein synthesis, phenylbutyrate, skeletal muscle

## Abstract

**New Findings:**

- **What is the central question of this study?**
  The compound sodium phenylbutyrate (PB) has been shown to promote branched‐chain amino acid (BCAA) catabolism, and as such has been proposed as a treatment for disorders with enhanced BCAA levels: does PB induce muscle protein catabolism by forcing BCAA degradation away from muscle protein synthesis and mechanistic target of rapamycin (mTOR) inhibition?
- **What is the main finding and its importance?**
  Accelerated BCAA catabolism using PB resulted in adverse effects related to mTOR signalling and muscle protein metabolism in skeletal muscle cells, which may limit its application in conditions where muscle wasting is a risk.

**Abstract:**

The compound sodium phenylbutyrate (PB) has been used for reducing ammonia in patients with urea cycle disorders and proposed as a treatment for disorders with enhanced branched‐chain amino acid (BCAA) levels, due to its effects on promoting BCAA catabolism. In skeletal muscle cells, we hypothesised that PB would induce muscle protein catabolism due to forcing BCAA degradation away from muscle protein synthesis and downregulating mechanistic target of rapamycin (mTOR). PB reduced medium BCAA and branched‐chain keto acid (BCKA) concentrations, while total cell protein (−21%; *P *< 0.001 vs. control) and muscle protein synthesis (−25%; *P *< 0.001 vs. control; assessed by measurement of puromycin incorporation into polypeptides) were decreased with PB. The regulator of anabolic pathways mTOR and its downstream components were impaired with PB treatment. The present results indicate that accelerated BCAA catabolism using PB resulted in adverse effects related to mTOR signalling and muscle protein metabolism, which may limit its application in settings where muscle wasting is a risk.

## Graphical abstract

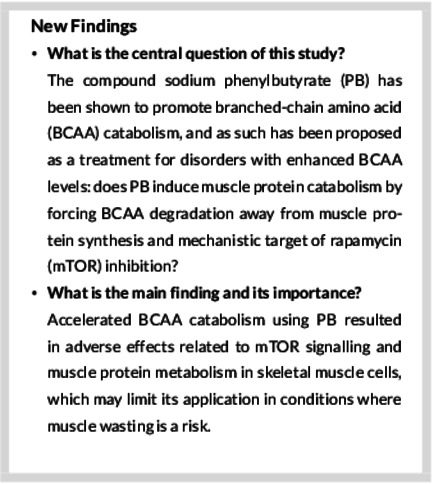

## 1 INTRODUCTION

The compound sodium phenylbutyrate (PB) has been successfully used as an ammonia‐lowering drug for patients with urea cycle disorders (UCDs), whereby nitrogen is disposed of in the urine in the form of phenylacetylglutamine (Brusilow, 1991). PB acts by scavenging ammonia using muscle as a surrogate for other failing organs, for example the liver. The therapeutic potential of PB has also been investigated in a wide range of conditions, including Huntington's disease, type 2 diabetes, cancer and sickle cell disease (Camacho et al., 2007; Collins et al., 1995; Gardian et al., 2005; Özcan et al., 2006). The exact mechanisms by which PB may exert beneficial effects across a wide range of disorders remain unclear, although one observed effect of PB treatment is decreased levels of endoplasmic reticulum (ER) stress (Kolb et al., 2015). Another observed consequence of PB treatment relates to its effect on branched‐chain amino acid (BCAA) metabolism, where studies have reported substantial decreases in circulating BCAA concentrations with PB administration in patients with UCD (Scaglia, Carter, O'Brien, & Lee, 2004). PB, which is converted into the active metabolite phenylacetate *in vivo*, is conjugated with glutamine to form phenylacetylglutamine, for excretion in the urine (Holeček, 2018). In patients with maple syrup urine disease, a disorder of BCAA metabolism that results in elevations of BCAAs in plasma, PB reduced circulating BCAAs and branched‐chain α‐keto acids (BCKAs), by increasing flux through the BCKA dehydrogenase complex (Brunetti‐Pierri et al., 2011).

Given the critical roles of BCAAs in the regulation of muscle protein metabolism, it is feasible that administration of PB will result in depletion of extracellular BCAAs, leading to downstream dysregulation of muscle protein synthesis (MPS) and/or breakdown, which could question its effectiveness as a treatment under certain conditions. Given the importance of BCAAs in essential processes, their depletion with PB could feasibly have adverse effects on the body, whereby such impairments in anabolic pathways could potentially exacerbate declines in muscle function in conditions already characterised by dysregulated protein metabolism, such as diabetes and cancer. In support of this, prior *in vitro* studies have demonstrated that PB treatment can result in muscle atrophy and impaired differentiation in C2C12 myotubes (Brown et al., 2018), but in contrast, did not blunt clenbuterol‐induced muscle growth *in vivo*. In another study, increased leucine oxidation and decreased protein synthesis were observed in *ex vivo* incubated rat muscles treated with PB (Holecek et al., 2017). However, again contrary to these findings, in diabetic rats, long‐term administration of PB decreased ubiquitin proteasome‐mediated protein degradation and atrophy in muscle (Reddy et al., 2019). As such, the effects of PB on muscle metabolism remain incompletely understood and in particular, how BCAA catabolism impacts signalling by mechanistic target of rapamycin (mTOR) – which is the molecular sensor of intracellular BCAAs.

Thus, the aims of the present study were to investigate the impact of PB on muscle protein metabolism. Using an *in vitro* murine skeletal muscle cell model, we assessed the impact of PB treatment on lowering extracellular BCAAs, and the subsequent effects on muscle protein homeostasis. We hypothesised that accelerating the catabolism of BCAA would result in a dysregulation of mTOR signalling and protein synthesis, resulting in cell protein catabolism.

## 2 METHODS

### 2.1 C2C12 cell culture experiments

Murine C2C12 myoblasts (passage 6–8; ECACC, Salisbury, UK) were cultured at 37°C and 5% CO_2_ in Dulbecco's modified Eagle medium/F‐12 (DMEM/F‐12, cat. no. 21041033; Thermo Fisher Scientific, Loughborough, UK) supplemented with 10% (v/v) fetal bovine serum (FBS) and 1% (v/v) antibiotic‐antimycotic solution (Sigma‐Aldrich, Dorset, UK). Cells were seeded onto six‐well plates (Nunclon™ Delta; Thermo Fisher Scientific) and when cells reached ∼90% confluency, differentiation was induced by replacing the medium with DMEM containing 2% (v/v) horse serum. Experiments were performed on day 4–5 post‐induction of differentiation, with a medium change performed every 48 h.

Cells were treated at the point of a medium change (using normal differentiation medium) with 10 mmol l^–1^ (in the range of previously used doses, e.g., Brown et al., 2018; Holecek et al., 2017) sodium PB for 6 or 24 h (*n* = 5–6 experimental/well replicates per treatment group). At the end of each time point, culture medium was collected and cells were harvested in homogenization buffer (50 mmol l^–1^ Tris–HCl, pH7.5, 1 mmol l^–1^ EDTA, 1 mmol l^–1^ EGTA, 10 mmol l^–1^ β‐glycerophosphate, 50 mmol l^–1^ NaF and a complete protease inhibitor cocktail tablet (Roche, West Sussex, UK)) for immunoblotting analysis and measurement of intracellular amino acid concentrations (see below). In separate experiments, cells were also harvested after 24 h in 0.3 mol l^–1^ NaOH for measurement of total protein, RNA and DNA. Trypan Blue staining and visual assessment was used to ensure treatments were not causing widespread adverse effects on cell viability. Cells were incubated with 0.4% (v/v) Trypan Blue for 1–2 min, then washed with PBS and visualised under a light microscope. Viability was assessed visually, that is, non‐viable cells were stained and live cells unstained.

### 2.2 Measurement of medium and intracellular amino acids and branched‐chain α‐keto acids

Cell culture medium (100 μl) with 10 μl internal standard (a mix of stable isotopically labelled amino acids) added, or isolated intracellular amino acids, was purified by passing through H^+^ Dowex resin columns and eluting into 2 mol l^–1^ NH_4_OH before being dried down. Amino acids were derivatised to their *tert*‐butyldimethylsilyl esters, and concentrations were determined using GC‐MS (Trace 1300‐ISQ; Thermo Fisher Scientific) with a standard curve of known concentrations. Intracellular amino acid concentrations were normalised against total cellular protein per well (using values from protein quantification prior to western blot analysis).

Medium concentrations of BCKAs (α‐ketoisocaproic acid, KIC; α‐keto‐β‐methylvaleric acid, KMV; and α‐ketoisovaleric acid, KIV) were determined by mixing 100 μl of cell culture medium with α‐ketovaleric acid as internal standard, deproteinizing with ice‐cold ethanol and drying down under nitrogen. The BCKAs were derivatised to quinoxalinol‐*tert*‐butyldimethylsilyl derivatives, and concentrations were determined using GC‐MS (Trace 1300‐ISQ; Thermo Fisher Scientific) with a standard curve of known concentrations of each BCKA.

### 2.3 Measurement of muscle protein synthesis

Muscle protein synthesis was determined in the 6‐h experiments using the surface sensing of translation (SUnSET) technique (Schmidt et al., 2009), which involves detection of the incorporation of puromycin (a tyrosyl‐tRNA analogue) into newly synthesised polypeptide chains. Puromycin (0.5 μmol l^–1^ final concentration) was added to the cells for the final 3 h of the 6‐h experiment. Immunoblotting (see below) was used to measure puromycin‐labelled peptides with a monoclonal puromycin antibody (12D10; EMD Millipore, Watford, UK).

### 2.4 Protein:DNA ratio assessments

Samples that were treated for 24 h with PB were harvested in 0.3 M NaOH for measurement of total alkaline‐soluble protein, RNA and DNA using the method described by Forsberg et al. (1991). In brief, samples were incubated at 37°C for 20 min and total protein was quantified using a NanoDrop™ 2000 (Thermo Fisher Scientific). Perchloric acid (1 mol l^–1^) was then added to each sample before incubation at 4°C for 30 min. Following centrifugation, the supernatant was quantified for RNA, then following removal of the supernatant, 2 mol l^–1^ perchloric acid was then added to the pellet and samples were incubated at 70°C for 1 h. Finally, DNA was quantified in the resultant supernatant.

### 2.5 Western blot analysis

Samples were lysed by repeatedly passing through gel‐loading pipette tips and lysates were centrifuged at 13,000 *g* for 10 min at 4°C. Protein from each sample (total 10 μg) was loaded onto 12% Criterion XT Bis‐Tris gels (Bio‐Rad Laboratories, Hemel Hempstead, UK) and samples were electrophoresed at 200 V for 1 h. Transfer to polyvinylidene difluoride membrane was performed at 100 V for 45 min, then membranes were blocked in 5% (w/v) milk for 1 h at room temperature. Primary antibody incubation was carried out overnight at 4°C with the following primary antibodies: mTOR Ser2448 (cat. no. 5536), ribosomal protein S6 kinase 1 (p70 S6K1) Thr389 (cat. no. 9234), eukaryotic translation initiation factor 4E binding protein 1 (4E‐BP1) Thr37/46 (cat. no. 2855) (from Cell Signaling Technology, Danvers, MA, USA). After primary antibody incubations, membranes were washed with 1× Tris‐buffered saline–Tween and incubated with anti‐rabbit horseradish peroxidase (HRP)‐conjugated secondary antibody (cat. no. 7074; Cell Signaling Technology; 1:2000 dilution) for 1 h at room temperature. After further washing with TBS–Tween, bands were detected using Chemiluminescent HRP substrate (EMD Millipore, Watford, UK) with a Chemidoc XRS imaging system (Bio‐Rad). Coomassie Brilliant Blue staining of the membrane was used for total protein normalisation and bands were quantified by densitometric analysis using Image Lab software (version 6; Bio‐Rad).

### 2.6 Statistical analyses

All statistical analyses were performed using GraphPad Prism 7 (GraphPad Software Inc., La Jolla, CA, USA). Descriptive statistics were produced to confirm normal distribution (accepted if *P *> 0.05). Data were analysed using Student's unpaired *t*‐test for two‐group comparisons, and where there were multiple time points, results were analysed by two‐way ANOVA with Tukey's *post hoc* test to locate specific differences. *P *< 0.05 was considered as statistically significant. All data are presented as box and whisker plots (where the median is the horizontal line, interquartile range is the box, minimum and maximum are the whiskers).

## 3 RESULTS

### 3.1 Amino acid and α‐keto acid concentrations with PB treatment in C2C12 myotubes

**FIGURE 1:**
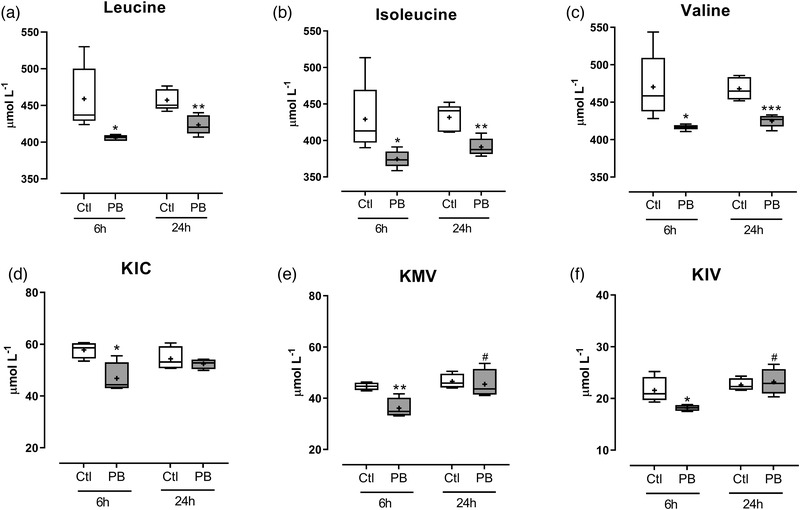
Medium branched‐chain amino acid and α‐keto acid concentrations in C2C12 myotubes following phenylbutyrate (PB) treatment. C2C12 myotubes were treated for 6 and 24 h with 10 mmol l^–1^ PB (*n* = 5 per group). Medium was analysed for concentrations of branched‐chain amino acids: leucine (a), isoleucine (b) and valine (c); and α‐keto acids: α‐ketoisocaproic acid (KIC; d), α‐keto‐β‐methylvaleric acid (KMV; e) and α‐ketoisovaleric acid (KIV; f). Data are presented as box and whisker plots (median is horizontal line; interquartile range is box; minimum and maximum are whiskers) and units are μmol l^–1^. *P < 0.05 vs. control (Ctl), **P < 0.01 vs. Ctl, ***P < 0.001 vs. Ctl. #*P *< 0.05 vs. 6 h

Treatment with 10 mmol l^–1^ PB caused a significant reduction in medium leucine (mean ± SEM 406 ± 2 vs. 459 ± 20 μmol l^–1^, *P *= 0.026 at 6 h; 423 ± 6 vs. 457 ± 6 μmol l^–1^, *P *= 0.0048 at 24 h), valine (417 ± 2 vs. 470 ± 20 μmol l^–1^, *P *= 0.027 at 6 h; 425 ± 4 vs. 468 ± 7 μmol l^–1^, *P *= 0.0005 at 24 h) and isoleucine (374 ± 5 vs. 428 ± 22 μmol l^–1^, *P *= 0.041 at 6 h; 391 ± 5 vs. 430 ± 7 μmol l^–1^, *P *= 0.0035 at 24 h) after 6 and 24 h, compared to untreated controls (Figure 1a–c). Treatment with 10 mmol l^–1^ PB also caused a significant reduction in medium KIC (46 ± 3 vs. 58 ± 2 μmol l^–1^, *P *= 0.017 vs. control (Ctl)), KMV (36 ± 2 vs. 45 ± 1 μmol l^–1^, *P *= 0.0064 vs. Ctl) and KIV (18 ± 1 vs. 22 ± 1 μmol l^–1^, *P *= 0.039 vs. Ctl) at 6 h relative to the control group, which was no different to control concentrations by 24 h (Figure 1d–f). For medium BCAA and KIC, there were no differences in concentrations between 6 and 24 h in either control or PB‐treated cells, but there was a relative increase in medium KIV (*P *= 0.02 vs. 6 h) and KMV (*P *= 0.026 vs. 6 h) between time points with PB treatment (Figure 1e, f).

**FIGURE 2:**
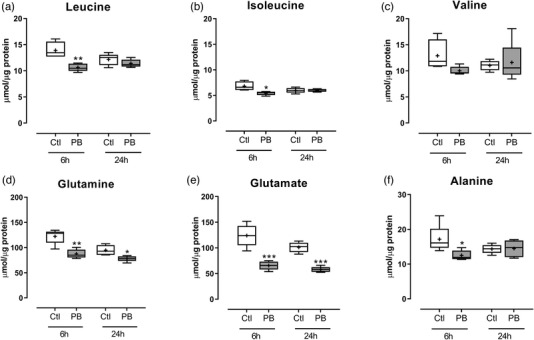
Intracellular amino acid concentrations in C2C12 myotubes following phenylbutyrate (PB) treatment. C2C12 myotubes were treated for 6 and 24 h with 10 mmol l^–1^ PB (*n* = 5 per group). Cells were analysed for intracellular concentrations of selected amino acids: leucine (a), isoleucine (b), valine (c), glutamine (d), glutamate (e) and alanine (f). Data are presented as box and whisker plots (median is horizontal line; interquartile range is box; minimum and maximum are whiskers) and units are μmol μg^–1^ protein. *P < 0.05 vs. control (Ctl), **P < 0.01 vs. Ctl, ***P < 0.001 vs. Ctl

Intracellular amino acid concentrations were assessed following PB treatment in C2C12 myotubes, normalised to cellular total protein. Treatment with 10 mmol l^–1^ PB had no effect on valine concentrations at 6 h, but there was a reduction in intracellular leucine (11 ± 0.3 vs. 14 ± 0.7 μmol μg^–1^ protein, *P *= 0.0042) and isoleucine (5.4 ± 0.2 vs. 6.8 ± 0.4 μmol μg^–1^ protein, *P *= 0.017) at 6 h (Figure 2a–c) compared to controls. After 24 h, intracellular BCAA concentrations were no different from controls (Figure 2a–c). There were no significant differences in intracellular amino acids across the time points measured (Figure 2). Concentrations of intracellular glutamine (87 ± 1.3 vs. 122 ± 2.7 μmol μg^–1^ protein, *P *< 0.0018), glutamate (66 ± 3.7 vs. 124 ± 9.5 μmol μg^–1^ protein, *P *= 0.0005) and alanine (13 ± 0.6 vs. 17 ± 1.8 μmol μg^–1^ protein, *P *= 0.036) were also decreased after 6‐h PB treatment (Figure 2d–f), relative to the control group, and while both glutamine and glutamate showed sustained decreases across all time points, by 24 h intracellular alanine levels were not different from those of control cells (Figure 2f).

### 3.2 Protein, RNA and DNA content, and changes in protein synthesis with PB treatment in C2C12 myotubes

**FIGURE 3:**
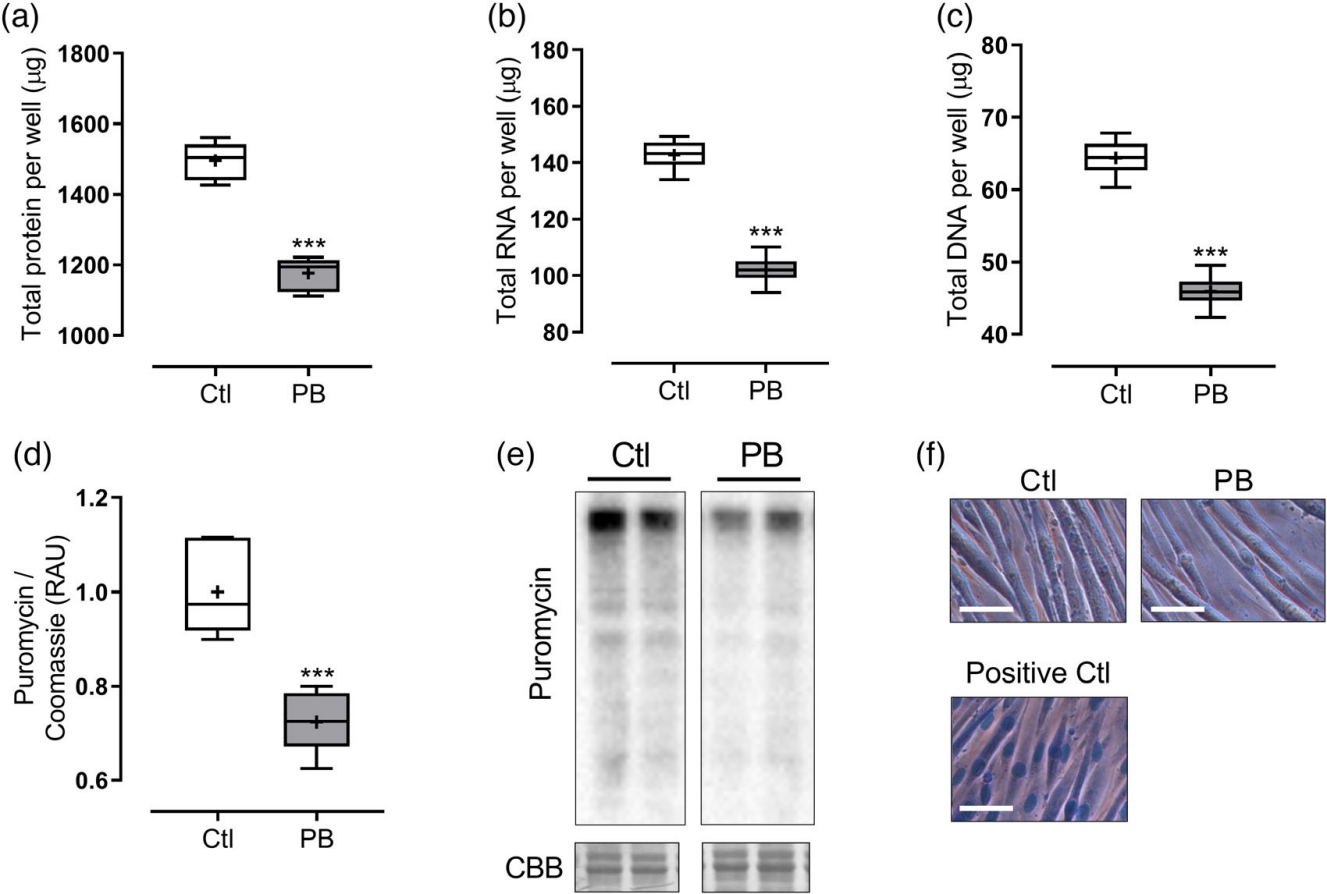
Total protein, RNA and DNA following PB treatment, and puromycin incorporation following PB treatment in C2C12 myotubes. C2C12 myotubes were treated for 24 h with 10 mmol l^–1^ PB (*n* = 6 per group). (a–c) Cells were analysed for total protein (a), RNA (b) and DNA (c) content. (d, e) In separate experiments, C2C12 myotubes were treated for 6 h with or without PB, with puromycin being added for the final 3 h. Cells were analysed for puromycin labelling of cellular polypeptides. Data are presented as box and whisker plots (median is horizontal line; interquartile range is box; minimum and maximum are whiskers). (f) Cells were stained after 24 h with Trypan Blue to assess changes in cell viability. Scale bars represent 100 μm. ***P < 0.001 vs. control (Ctl). CBB, Coomassie Brilliant Blue

To evaluate the impact of PB on muscle anabolic signalling and aspects of cell size, total protein, RNA and DNA content was measured following PB administration, as well as selected regulators of anabolic pathways. Total alkaline‐soluble protein, RNA and DNA content per well were all decreased following 24‐h PB treatment (respectively 1177 ± 19 vs. 1496 ± 22 μg, *P *< 0.0001; 45.9 ± 0.9 vs. 64.1 ± 0.8 μg, *P *< 0.0001; and 17.3 ± 0.5 vs. 21.4 ± 0.5 μg, *P *< 0.0001; Figure 3a–c) in comparison to controls. Protein synthesis, measured by puromycin incorporation into cellular protein, was significantly decreased following 6‐h PB treatment (*P *= 0.0002 vs. Ctl; Figure 3d, e). An absence of staining with Trypan Blue after 24 h confirmed there was no decrease in cell viability with PB treatment (Figure 3f).

**FIGURE 4:**
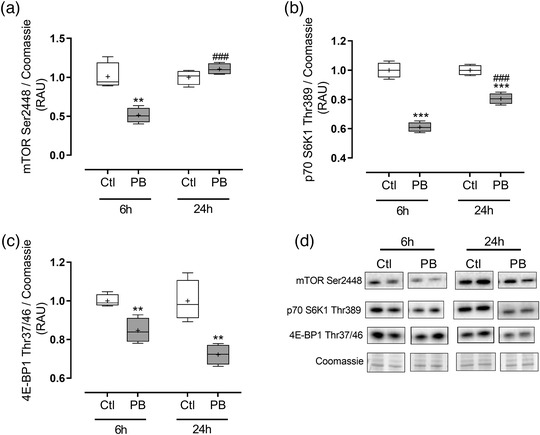
Anabolic‐related signalling changes in C2C12 myotubes following phenylbutyrate (PB) treatment. C2C12 myotubes were treated for 6 and 24 h with 10 mmol l^–1^ PB (*n* = 5 per group). Cells were analysed for phosphorylation of mechanistic target of rapamycin (mTOR; Ser2448) (a), ribosomal protein S6 kinase 1 (p70 S6K1; Thr389) (b) and eukaryotic translation initiation factor 4E‐binding protein 1 (4E‐BP1; Thr37/46) (c). Data are presented as box and whisker plots (median is horizontal line; interquartile range is box; minimum and maximum are whiskers). **P < 0.01 vs. control (Ctl), ***P < 0.001 vs. Ctl. ###*P *< 0.001 vs. 6 h. RAU, relative arbitrary units

In terms of anabolic signalling pathways, PB resulted in a decrease in mTOR phosphorylation at 6 h compared with untreated cells (0.5 ± 0.04‐fold; *P *= 0.0024), which was no different from control by 24 h (Figure 4a). Phosphorylated p70 S6K1 (Thr389) was significantly decreased with PB at both 6 h (*P *< 0.0001 vs. Ctl) and 24 h (*P *= 0.0002 vs. Ctl; Figure 4b). For both mTOR and p70 S6K1, there were relative increases in phosphorylation in the PB‐treated cells at 24 h versus 6 h (both *P *< 0.0001 vs. 6 h; Figure 4a, b). Finally, phosphorylated 4E‐BP1 (Thr37/46) was also decreased at both time points (*P *= 0.0052 vs. Ctl at 6 h; *P *= 0.0033 vs. Ctl at 24 h; Figure 4c) with PB, relative to the control group.

## 4 DISCUSSION

Administration of PB has been used as an ammonia‐lowering drug for patients with UCD (Brusilow, 1991), but depletion of circulating BCAA has been observed in these patients (Scaglia et al., 2004), and PB has directly been shown to activate the BCKA dehydrogenase complex (Brunetti‐Pierri et al., 2011). Given the importance of BCAAs as critical substrates and regulators of MPS (Kimball & Jefferson, 2001), this effect of PB could have negative impacts on protein metabolism in muscle. For certain conditions characterised by underlying dysregulation of protein metabolism such as diabetes and cancer, these declines in BCAAs could potentially further negatively impact on anabolic processes in muscle, exacerbating declines in muscle function and health. It is important therefore to investigate the molecular mechanisms by which PB exerts its effects on muscle, where there is currently little insight available. We hypothesised that increasing BCAA catabolism using the compound PB would result in an mTOR‐associated dysregulation of protein homeostasis, resulting in cellular protein catabolism. We observed that while murine C2C12 myotubes treated with PB had lowered medium and cellular BCAA concentrations, prolonged treatment resulted in impairments in protein metabolism, which appeared to be, at least partly, due to reduced activity of mTOR‐regulated anabolic signalling and MPS.

During time‐course experiments, treatment with the compound PB (as expected from previous studies; Holecek et al., 2017) reduced medium concentrations of BCAAs after 6 h post‐treatment. BCKAs were also decreased in the medium 6 h post‐treatment, indicating that intracellular BCAAs were being metabolised at least beyond BCAA transaminase (BCAT), the first enzyme in the BCAA catabolism pathway. Catabolism of the BCAAs also impacted other amino acids intracellularly, with decreases in glutamine, glutamate and alanine following 6‐h treatment. Medium concentrations of glutamine and glutamate also followed similar decreases with PB treatment (data not shown). It has been proposed that PB results in depletion of glutamine, through formation of phenylacetylglutamine (Holeček, 2018). Studies have previously described that glutamine deficiency can have negative impacts on muscle protein metabolism (Holecek & Sispera, 2014), suggesting the declines in both cellular BCAA and glutamine may have been responsible for the impairments in muscle protein metabolism with PB administration. One limitation of the present study was that we did not measure phenylacetate levels following PB treatment and so cannot determine whether this conversion is required for its activity. However, the observed changes in BCAA and cellular glutamine support the chemistry of PB action in our present model. Overall, the model used in the present study produced the expected effects on BCAA catabolism, allowing us to directly assess the impact of decreased cellular BCAAs and glutamine on the regulation of protein metabolism in skeletal muscle cells.

Prolonged PB treatment (24 h) also resulted in decreased total cellular protein with the dose of PB used (10 mmol l^–1^), which could have reflected not only reduced anabolism but also potentially a protein catabolic response to the compound. There were also reductions in total cellular RNA and DNA with PB treatment, suggesting myotube loss may have occurred. Previous studies have reported atrophic effects of PB treatment in muscle (Brown et al., 2018), though whether this is a direct result of the depletion in extracellular BCAAs remains to be determined. Since there were impairments in mTOR activity with PB, it is feasible that additional signalling pathways were altered, including induction of autophagy, since mTOR is a key regulator of autophagy regulation via alteration of unc‐51‐like kinase 1/2 (Ulk1/2) kinase activity (Alers et al., 2012). The compound PB has also been shown to influence other biological pathways, including modulation of ER stress and inhibition of histone deacetylases (Khan et al., 2017). Future work should aim to uncover whether targeting BCAA catabolism using different approaches (e.g. using an inhibitor of BCKA dehydrogenase kinase; Zhou et al., 2019), would result in the observed adverse effects on protein metabolism, as well as explore the downstream impact of mTOR inhibition on activation of catabolic processes such as autophagy.

In terms of the effects of PB on MPS (measured in this study by assessing labelling of cellular protein with puromycin for the final 3 h of treatment), there was a significant decline with PB. This is consistent with previous observations (Brown et al., 2018; Holecek et al., 2017), and whether these changes are a direct result of reduced BCAA levels (i.e. secondary to reduced amino acid sensing from substrate shortage) remains to be evaluated. Future work could focus on inhibiting BCAT activity in cells to examine how this might alleviate the inhibition on MPS, although recent studies have suggested specific knockdown of BCAT2 inhibited myotube formation *in vitro* (Dhanani et al., 2019). We also assessed molecular markers that could be responsible for mediating these impairments in MPS; observing decreases in mTOR Ser2448 phosphorylation with 6‐h PB treatment, as well as reduced phosphorylation (i.e. reduced activation) of downstream regulators of translation initiation (p70 S6K1 and 4E‐BP1), which were still impaired after 24 h. Multiple amino acid‐sensing proteins have been identified, including the recombination‐activating gene (RAG) and Sestrin family of proteins, and proposed as important upstream regulators of mTOR activity (Shimobayashi & Hall, 2016). It is possible that one or more of these amino acid sensing pathways were responsible for the impaired mTOR activation in the present study, secondary to BCAA and/or glutamine depletion by PB.

In conclusion, we demonstrate that incubation with PB accelerated *in vitro* BCAA catabolism in muscle cells, and sustained treatment with PB resulted in impairments in protein metabolism, at least partly, due to an impairment in mTOR‐signalling and MPS. The findings contribute further insight into the impact of PB on muscle protein balance and suggest that adverse effects on muscle protein metabolism may be a side effect when PB is used clinically.

## COMPETING INTERESTS

None declared.

## AUTHOR CONTRIBUTIONS

K.S., II, B.E.P., P.J.A. and D.J.W. contributed to conception or design of the work. H.C., K.S., P.J.A. and D.J.W. contributed to data acquisition, analysis or interpretation of the work. All authors contributed to drafting and revising of the manuscript. All authors approved the final version of the manuscript and agree to be accountable for all aspects of the work in ensuring that questions related to the accuracy or integrity of any part of the work are appropriately investigated and resolved. All persons designated as authors qualify for authorship, and all those who qualify for authorship are listed.

## Data Availability

The data that support the findings of this study are available from the corresponding author upon reasonable request.

## References

[eph12927-bib-0001] Alers, S. , Loffler, A. S. , Wesselborg, S. , & Stork, B. (2012). Role of AMPK‐mTOR‐Ulk1/2 in the regulation of autophagy: Cross talk, shortcuts, and feedbacks. Molecular and Cellular Biology, 32, 2–11. 22025673 10.1128/MCB.06159-11 PMC3255710

[eph12927-bib-0002] Brown, D. M. , Jones, S. , Daniel, Z. C. T. R. , Brearley, M. C. , Lewis, J. E. , Ebling, F. J. P. , Parr, T. , & Brameld, J. M. (2018). Effect of sodium 4‐phenylbutyrate on Clenbuterol‐mediated muscle growth. PLoS One, 13, e0201481. 30052661 10.1371/journal.pone.0201481 PMC6063449

[eph12927-bib-0003] Brunetti‐Pierri, N. , Lanpher, B. , Erez, A. , Ananieva, E. A. , Islam, M. , Marini, J. C. , Sun, Q. , Yu, C. , Hegde, M. , Li, J. , Wynn, R. M. , Chuang, D. T. , Hutson, S. , & Lee, B. (2011). Phenylbutyrate therapy for maple syrup urine disease. Human Molecular Genetics, 20, 631–640. 21098507 10.1093/hmg/ddq507 PMC3024040

[eph12927-bib-0004] Brusilow, S. W. (1991). Phenylacetylglutamine may replace urea as a vehicle for waste nitrogen excretion. Pediatric Research, 29, 147–150. 2014149 10.1203/00006450-199102000-00009

[eph12927-bib-0005] Camacho, L. H. , Olson, J. , Tong, W. P. , Young, C. W. , Spriggs, D. R. , & Malkin, M. G. (2007). Phase I dose escalation clinical trial of phenylbutyrate sodium administered twice daily to patients with advanced solid tumors. Investigational New Drugs, 25, 131–138. 17053987 10.1007/s10637-006-9017-4

[eph12927-bib-0006] Collins, A. F. , Pearson, H. A. , Giardina, P. , McDonagh, K. T. , Brusilow, S. W. , & Dover, G. J. (1995). Oral sodium phenylbutyrate therapy in homozygous β thalassemia: A clinical trial. Blood, 85, 43–49. 7528572

[eph12927-bib-0007] Dhanani, Z. N. , Mann, G. , & Adegoke, O. A. J. (2019). Depletion of branched‐chain aminotransferase 2 (BCAT2) enzyme impairs myoblast survival and myotube formation. Physiological Reports, 7, e14299. 31833233 10.14814/phy2.14299 PMC6908738

[eph12927-bib-0008] Forsberg a, M. , Nilsson, E. , Werneman, J. , Bergström, J. , & Hultman, E. (1991). Muscle composition in relation to age and sex. Clinical Science, 81, 249–256. 1716189 10.1042/cs0810249

[eph12927-bib-0009] Gardian, G. , Browne, S. E. , Choi, D. K. , Klivenyi, P. , Gregorio, J. , Kubilus, J. K. , Ryu, H. , Langley, B. , Ratan, R. R. , Ferrante, R. J. & Beal, M. F. (2005). Neuroprotective effects of phenylbutyrate in the N171‐82Q transgenic mouse model of Huntington's disease. Journal of Biological Chemistry, 280, 556–563. 15494404 10.1074/jbc.M410210200

[eph12927-bib-0010] Holeček, M. (2018). Branched‐chain amino acids in health and disease: Metabolism, alterations in blood plasma, and as supplements. Nutrition and Metabolism, 15, 33. 29755574 10.1186/s12986-018-0271-1 PMC5934885

[eph12927-bib-0011] Holecek, M. , & Sispera, L. (2014). Glutamine deficiency in extracellular fluid exerts adverse effects on protein and amino acid metabolism in skeletal muscle of healthy, laparotomized, and septic rats. Amino Acids, 46, 1377–1384. 24609272 10.1007/s00726-014-1701-7

[eph12927-bib-0012] Holecek, M. , Vodenicarovova, M. , & Siman, P. (2017). Acute effects of phenylbutyrate on glutamine, branched‐chain amino acid and protein metabolism in skeletal muscles of rats. Internatiional Journal of Experimental Pathology, 98, 127–133. 10.1111/iep.12231 PMC5573773 28621016

[eph12927-bib-0013] Khan, S. , Komarya, S. K. , & Jena, G. (2017). Phenylbutyrate and β‐cell function: Contribution of histone deacetylases and ER stress inhibition. Epigenomics, 9, 711–720. 28470097 10.2217/epi-2016-0160

[eph12927-bib-0014] Kimball, S. R. , & Jefferson, L. S. (2001). Regulation of protein synthesis by branched‐chain amino acids. Current Opinion in Clinical Nutrition and Metababolic Care, 4, 39–43. 10.1097/00075197-200101000-00008 11122558

[eph12927-bib-0015] Kolb, P. S. , Ayaub, E. A. , Zhou, W. , Yum, V. , Dickhout, J. G. , & Ask, K. (2015). The therapeutic effects of 4‐phenylbutyric acid in maintaining proteostasis. International Journal of Biochemistry and Cell Biology, 61, 45–52. 25660369 10.1016/j.biocel.2015.01.015

[eph12927-bib-0016] Özcan, U. , Yilmaz, E. , Özcan, L. , Furuhashi, M. , Vaillancourt, E. , Smith, R. O. , Görgün, C. Z. , & Hotamisligil, G. S. (2006). Chemical chaperones reduce ER stress and restore glucose homeostasis in a mouse model of type 2 diabetes. Science, 313, 1137–1140. 16931765 10.1126/science.1128294 PMC4741373

[eph12927-bib-0017] Reddy, S. S. , Shruthi, K. , Joy, D. , & Reddy, G. B. (2019). 4‐PBA prevents diabetic muscle atrophy in rats by modulating ER stress response and ubiquitin‐proteasome system. Chemico‐Biological Interactions, 306, 70–77. 30980806 10.1016/j.cbi.2019.04.009

[eph12927-bib-0018] Scaglia, F. , Carter, S. , O'Brien, W. E. , & Lee, B. (2004). Effect of alternative pathway therapy on branched chain amino acid metabolism in urea cycle disorder patients. Molecual Genetics and Metabolism, 81, 79–85. 10.1016/j.ymgme.2003.11.017 15050979

[eph12927-bib-0019] Schmidt, E. K. , Clavarino, G. , Ceppi, M. , & Pierre, P. (2009). SUnSET, a nonradioactive method to monitor protein synthesis. Nature Methods, 6, 275–277. 19305406 10.1038/nmeth.1314

[eph12927-bib-0020] Shimobayashi, M. , & Hall, M. N. (2016). Multiple amino acid sensing inputs to mTORC1. Cell Research, 26, 7–20. 26658722 10.1038/cr.2015.146 PMC4816134

[eph12927-bib-0021] Zhou, M. , Shao, J. , Wu, C. Y. , Shu, L. , Dong, W. , Liu, Y. , Chen, M. , Wynn, R. M. , Wang, J. , Wang, J. , Gui, W.‐J. , Qi, X. , Lusis, A. J. , Li, Z. , Wang, W. , Ning, G. , Yang, X. , Chuang, D. T. , Wang, Y. , & Sun, H. (2019). Targeting BCAA catabolism to treat obesity‐associated insulin resistance. Diabetes, 68, 1730–1746. 31167878 10.2337/db18-0927 PMC6702639

